# Stabilization of G protein-coupled receptors by point mutations

**DOI:** 10.3389/fphar.2015.00082

**Published:** 2015-04-20

**Authors:** Franziska M. Heydenreich, Ziva Vuckovic, Milos Matkovic, Dmitry B. Veprintsev

**Affiliations:** ^1^Laboratory of Biomolecular Research, Paul Scherrer InstitutVilligen, Switzerland; ^2^Department of Biology, ETH ZürichZürich, Switzerland

**Keywords:** G protein-coupled receptors, GPCRs, conformational thermostabilization, protein engineering, alanine scanning, pharmacology, stabilizing mutations

## Abstract

G protein-coupled receptors (GPCRs) are flexible integral membrane proteins involved in transmembrane signaling. Their involvement in many physiological processes makes them interesting targets for drug development. Determination of the structure of these receptors will help to design more specific drugs, however, their structural characterization has so far been hampered by the low expression and their inherent instability in detergents which made protein engineering indispensable for structural and biophysical characterization. Several approaches to stabilize the receptors in a particular conformation have led to breakthroughs in GPCR structure determination. These include truncations of the flexible regions, stabilization by antibodies and nanobodies, fusion partners, high affinity and covalently bound ligands as well as conformational stabilization by mutagenesis. In this review we focus on stabilization of GPCRs by insertion of point mutations, which lead to increased conformational and thermal stability as well as improved expression levels. We summarize existing mutagenesis strategies with different coverage of GPCR sequence space and depth of information, design and transferability of mutations and the molecular basis for stabilization. We also discuss whether mutations alter the structure and pharmacological properties of GPCRs.

## Introduction

G protein-coupled receptors (GPCRs) are integral membrane proteins that play a central role in signaling pathways being key intermediaries between external stimuli and the intracellular signaling cascades. They consist of a single polypeptide chain with seven transmembrane domains, an extracellular N-terminus and an intracellular C-terminus. They are only found in eukaryotes with over 800 different GPCRs identified in humans so far, 400 of which are non-olfactory receptors (Fredriksson et al., 2003; Bjarnadóttir et al., 2006). GPCRs regulate vision, smell and taste as well as many other physiological processes (Pierce et al., 2002). They interact with photons, proteins, hormones, neurotransmitters, and small molecules. A GPCR may bind several different ligands, which induce distinct conformational changes (Deupi and Kobilka, 2007, 2010; Kobilka and Deupi, 2007). Such changes lead to different signaling modes and altered biological responses.

Binding of the ligand on the extracellular side of a GPCR elicits a conformational change that extends to the intracellular surface of the receptor and results in activation of heterotrimeric G proteins by exchange of GDP for GTP in the Gα subunit, followed by dissociation of Gα and Gβγ which results in a change in intracellular second messengers levels (Gilman, 1987; Oldham and Hamm, 2008). Subsequent hydrolysis of the GTP returns the G protein to its inactive state.

For many years, only cellular and biochemical studies have provided insight into GPCR function. Crystallization and structure determination, however, lagged behind, which is due to low expression levels and the poor stability of GPCRs in detergents (Warne et al., 2009). For a long time, GPCRs could not be crystallized or the produced crystals did not diffract well enough for structure determination. The first structure of a GPCR, the structure of bovine rhodopsin, was determined in 2000 (Palczewski et al., 2000). Then it took until 2007 to get the structure of another GPCR, human β_2_ adrenergic receptor (Cherezov et al., 2007; Rasmussen et al., 2007; Rosenbaum et al., 2007).

To date, crystallization and subsequent structural characterization of GPCRs requires extensive protein engineering (Kobilka and Schertler, 2008; Blois and Bowie, 2009; Tate and Schertler, 2009; Chun et al., 2012; Tate, 2012; Bertheleme et al., 2013; Scott et al., 2013). The only exceptions are bovine and squid rhodopsin, which could be extracted from their native source, where they are present in high abundance. They do not require extensive purification and are stable in detergents (Palczewski et al., 2000; Murakami and Kouyama, 2008). On the other hand, the determination of the crystal structure of recombinantly produced bovine rhodopsin in its partially deglycosylated form required a high factor of purification and the introduction of a stabilizing disulfide bond between N terminus and extracellular loop 3 (Standfuss et al., 2007). Current techniques for GPCR studies include protein engineering and conformational stabilization by replacement of flexible loops, shortening of N- or C-termini or introduction of stabilizing mutations. Fusion partners such as T4 lysozyme (T4L) or thermostabilized cytochrome b_562_RIL (BRIL) have been used to stabilize GPCRs, reduce flexibility and facilitate their crystallization (Chun et al., 2012). Besides the stabilization of the receptor, the important factors for the increase in the number of solved structures are the addition of antibody F_ab_ fragments, nanobodies and the use of lipidic cubic phase crystallization (Rasmussen et al., 2007, 2011a,b; Weis and Kobilka, 2008).

While there is a set of commonly used fusion partners available for stabilization of GPCRs, a new set of stabilizing mutations had to be identified for each receptor so far. Stabilizing mutations have been found using a variety of techniques including random error-prone PCR or all-vs.-all mutation combined with evolutionary approaches (Sarkar et al., 2008; Dodevski and Plückthun, 2011; Schlinkmann and Plückthun, 2013; Scott and Plückthun, 2013; Scott et al., 2014) and systematic alanine scanning mutagenesis (Magnani et al., 2008; Serrano-Vega et al., 2008; Shibata et al., 2009; Robertson et al., 2011; Hollenstein et al., 2013; Doré et al., 2014; Hirozane et al., 2014). Most commonly used assays for conformational, thermal and detergent stability include radio-ligand binding assays, fluorescence size-exclusion chromatography (FSEC) and fluorescence activated cell sorting (FACS) with fluorescently-labeled ligands. Recently, computational prediction approaches have been suggested. They were used to validate existing experimental alanine scanning data, but have yet to be verified for new receptors (Chen et al., 2012b; Bhattacharya et al., 2014).

## Methods for stabilization of non-GPCR proteins

Stabilization by mutagenesis is widely employed for soluble proteins and includes stabilization of industrial enzymes, antibodies, and fluorescent proteins (Ahern et al., 1987; Arase et al., 1993; Amin et al., 2004; Pédelacq et al., 2006; reviewed in Nielsen and Borchert, 2000; Wörn and Plückthun, 2001; Ó'Fágáin, 2003). These methods include semi-rational protein design using one or multiple homologs of the target protein as a source of possible thermostabilizing variants, random mutagenesis and scanning mutagenesis.

Influence of single amino acid changes on protein stability has been studied for barnase where mutations were shown to change protein stability to different degrees ranging from +1.1 kcal/mol to −1.1 kcal/mol. This stabilization energy corresponds to a change in thermostability of approximately 3°C for this protein. Stabilization and destabilization are connected to a change in hydrophobic surface buried in the folded state as well as a loss or gain of favorable interactions. Several stabilizing mutations decreased flexibility and thereby increased thermostability (Serrano et al., 1993). Semi-rational as well as random mutagenesis was tested for p53, resulting in higher stability and increased half-life (Nikolova et al., 1998; Matsumura and Ellington, 1999). Interestingly, the final quadruple mutant from semi-rational protein design and the triple mutant from random mutagenesis share two mutations. Diacylglycerol kinase (DGK), an integral membrane protein from *Escherichia coli*, has been stabilized by two different approaches: random PCR mutagenesis and introduction of cysteine mutants (Lau et al., 1999; Zhou and Bowie, 2000). The approaches included semi-rational protein design using one or multiple homologs of the target protein as a source of possible thermostabilizing variants, use of disease-rescue mutations, random mutagenesis and scanning mutagenesis. Analysis of several studies on insertion of mutations indicated that approximately 10% of randomly inserted mutations stabilized the protein (Bowie, 2001).

## Stabilization of GPCRs by point mutations and their combination

Currently there is no clear design strategy for stabilization of GPCRs by mutations. Therefore, stabilizing mutations have to be identified experimentally by testing many different point mutations in either one-by-one or by ensemble evolutionary approaches. When single mutations are identified, they can be combined to further increase the thermostability of the protein. The process of combining the mutations is also experimental because the effects of individual mutations are not always additive and the structural basis for stabilization is not necessarily obvious. However, some general observations have been formulated. Effects of replacing residues which are neighbors in sequence or structure usually do not lead to a further increase and may even decrease the stability of the protein, as effects of single mutations may cancel each other when combined. Combinations of non-neighboring mutations may lead to a further stabilization, though the increase in stability is usually smaller than the summed up stabilization effects conferred by single mutations (Magnani et al., 2008; Serrano-Vega et al., 2008; Shibata et al., 2009, 2013; Lebon et al., 2011a). It has been observed that mutations stabilizing the agonist-bound state are more difficult to combine as they may stabilize slightly different active conformations. In addition, active conformations are more open on the intracellular side which may be more difficult to stabilize compared to a more compact, less dynamic inactive state (Magnani et al., 2008).

## Alanine and leucine scanning mutagenesis

The major approach to finding single stabilizing mutations is an exchange of all amino acids of the GPCR one by one. At the current level of technology, all-vs.-all mutations would lead to too many protein variants for individual analysis. Therefore, amino acids are exchanged for only one amino acid, commonly alanine. Alanine itself is exchanged for leucine. Alanine and leucine are the amino acids of choice due to their high helix propensity (Horovitz et al., 1992; Blaber et al., 1993) and the low probability of steric hindrances due to their small size. Additionally, both alanine and leucine show a high occurrence in α-helical membrane proteins (Eilers et al., 2002).

Alanine scanning has been used for stabilization of turkey β1-adrenergic receptor in the inactive conformation (Serrano-Vega et al., 2008; Warne et al., 2009), human adenosine A_2A_ receptor (Magnani et al., 2008; Lebon et al., 2011a; Robertson et al., 2011), and rat neurotensin receptor NTR1 with and without agonist bound (Shibata et al., 2009, 2013). Additional leucine scanning mutagenesis combined with salt-bridge engineering improved the turkey β1-adrenergic receptor further (Miller and Tate, 2011). In addition, the structures of the class C metabotropic glutamate receptor 5 transmembrane domain, class B corticotropin-releasing factor receptor 1 and adenosine A_2A_ receptor in complex with different ligands were solved using conformationally thermostabilized receptors generated by alanine scanning (Doré et al., 2011, 2014; Hollenstein et al., 2013). Finally, a recent mutagenesis study based on alanine/valine scanning and exchange of tyrosine for alanine and phenylalanine lead to thermostabilized FFA receptor 1 (Hirozane et al., 2014).

While alanine or leucine scanning could technically be done on a wild type receptor, this has not been tried for GPCRs so far. In turkey β1-adrenergic receptor N- and C-termini were truncated and a mutation, C116L, was inserted. This led to an increased expression level and improved solubilization which might have been hindered by the extended C-terminal domain (Warne et al., 2003; Serrano-Vega et al., 2008; Miller and Tate, 2011). In adenosine A_2A_ receptor, the 96 C-terminal residues were deleted in order to prevent proteolytic degradation upon solubilization (Weiß and Grisshammer, 2002; Magnani et al., 2008; Lebon et al., 2011a). Rat neurotensin receptor NTR1 was N-terminally truncated to start at T43 (White et al., 2004; Shibata et al., 2009, 2013).

Single alanine/leucine mutants have been expressed in *Escherichia coli*, the cells were lysed and the receptors solubilized using different detergents. Alanine scanning or generation of stabilized receptors based on HEK293T cell expression was done for M_1_ muscarinic receptor, metabotropic glutamate receptor 5 and corticotropin-releasing factor receptor 1 (Robertson et al., 2011; Hollenstein et al., 2013; Doré et al., 2014). Thermostability has been assessed by incubation of the mutant receptors at the apparent melting temperature of the wild type and measurement of retained ligand binding activity. It has to be noted that the melting temperature varies significantly with the experiment design, especially the length of time for which the receptor is incubated at elevated temperatures and the detergent used for the assay. Short chain detergents such as octyl glucoside (OG) are more destabilizing since they create smaller micelles around the protein which is favorable for crystallization. Longer chain detergents such as dodecyl maltoside (DDM) preserve the native structure and activity of the protein better but are not suitable for vapor-diffusion crystallization. The use of detergents is further discussed in several other papers (Rigaud et al., 1995; Kragh-Hansen et al., 1998; Seddon et al., 2004; Privé, 2007; Linke, 2009; Lichtenberg et al., 2013; Meyer et al., 2015).

Alanine scanning for stabilization of agonist- or antagonist-bound state may benefit from the presence of ligand in the heating step. The presence of the ligand in the heating step resulted in a different pool of stabilizing mutants for adenosine A_2A_ receptor (Lebon et al., 2011a). These mutations have shown a higher thermostabilization than the mutants identified in the assay in absence of ligand (Magnani et al., 2008). Presence of agonist may favor selection of mutations which stabilize the agonist-bound state without requiring a stable inactive state. To develop a receptor which tolerates detergents in the apo-state for ligand-affinity purification, ligand was omitted in the heating step (Shibata et al., 2009). This ensures that the selected mutations stabilize the apo-state.

A likely explanation for the identification of different mutants depending on assay format is that ligands stabilize a certain conformation of the receptor. In the apo-state, however, the receptor switches between several inactive and active conformations (Bockenhauer et al., 2011). One could argue that agonist alone is not sufficient to induce the fully active state in diffusible-ligand activated GPCRs, at least for the β2-adrenergic receptor (Deupi and Kobilka, 2007; Kobilka and Deupi, 2007; Yao et al., 2009), but so far selection in the presence of G protein has not been reported.

Mutants should show a significant increase in residual activity after heating, usually 65–75% activity where the wild type retains 50% activity. However, higher thermostability does not necessarily correlate with higher protein expression. Therefore, a minimal expression limit should be set.

Although used in the initial screen, alanine and leucine may not be the most stabilizing amino acids. Exchange of the alanine or leucine for different amino acids of varying size and charge has been advantageous for turkey β1-adrenergic receptor. One third of the mutations could be further improved by exchange for different amino acids (Serrano-Vega et al., 2008).

More commonly, the thermostabilizing mutations are combined by either PCR with random mixes of primers leading to a random combination of mutations (Magnani et al., 2008; Serrano-Vega et al., 2008), or by design. In the design approach, the most thermostabilizing mutant is used as a base (a new pseudo wild-type) and the other mutations are combined with the base mutant, leading to the identification of the best double mutant (Lebon et al., 2011a). The pool of additive mutations from the first round is then combined with the best double mutant on a one-by-one basis, until sufficient stabilization for subsequent crystallization attempts is achieved (Lebon et al., 2011a).

Alanine scanning combined with radio-ligand binding does not only identify stabilizing mutations but leads to a conformationally and thermally stabilized construct which can then be used for crystallization and subsequent structure determination. Turkey β1-adrenergic receptor was improved by insertion of six point mutations which increased the apparent melting temperature of the receptor by 21°C. Addition of antagonist increased the melting temperature of the stabilized receptor by another 2°C while the T_m_ of non-stabilized receptor was increased by 6°C. This suggests that the receptor is conformationally thermostabilized in an inactive state, which is further confirmed by decreased affinity for agonists while the antagonist binding stays unaffected (Serrano-Vega et al., 2008). The mutation of the palmitoylation site C358^8.59^A was done to avoid the possibility of heterogeneous palmitoylation (Warne et al., 2008, 2009). Stabilization of human adenosine A_2A_ receptor led to an increased thermostability of 9°C for the agonist-stabilized receptor and 17°C for the antagonist-stabilized receptor in their ligand-bound forms (Magnani et al., 2008); a second alanine scanning of the receptor led to a stability increase of 21.5°C in presence of the agonist NECA (Lebon et al., 2011a). Rat neurotensin 1 receptor stability was increased by 13°C in presence and 17°C in absence of ligands as compared to the base construct (Shibata et al., 2009). The M1 muscarinic receptor stability was increased by 18°C (Robertson et al., 2011). All receptors showed increased stability in short-chain detergents, which are considered harsher than long-chain detergents. For adenosine A_2A_ receptor, the final construct was independent of the lipid cholesteryl hemisuccinate (CHS) (Magnani et al., 2008; Serrano-Vega et al., 2008; Shibata et al., 2009; Robertson et al., 2011; Brueckner et al., 2013).

## Directed evolution approaches

An alternative to alanine scanning for thermostabilization of receptors is directed evolution. The main difference between scanning mutagenesis and directed evolution is the number of mutations which can be screened using evolution methods; while alanine scanning provides information on all generated mutations (usually 300–2000), directed evolution is used to screen more than a million mutations, but only selects the most stable and/or best expressing mutants. Directed evolution systems have been previously used to increase functional expression at the cell surface and stability in detergents (Sarkar et al., 2008; Dodevski and Plückthun, 2011; Schlinkmann et al., 2012a; Scott and Plückthun, 2013). All methods are based on selection of many GPCR variants harboring single point-mutations for increased expression at the cell surface or stability in detergents. *E. coli* is the host of choice for evolutionary methods due to its transformation efficiency which allows rapid screening of millions of mutants. However, all methods require a high-affinity fluorescently-labeled ligand for selection of GPCR variants with higher functional expression or stability.

Libraries of receptor variants can be generated by error-prone PCR. Libraries are transformed and expressed in the inner membrane of *E. coli*, the outer membrane is permeabilized to allow binding of fluorescently-labeled ligand to receptors. The cells are sorted by fluorescence -activated cell sorting (FACS), usually the 1% highest fluorescent cells are selected and multiple rounds of FACS sorting may be used. The cell sorting allows selection for the highest expression level at the cell surface without selecting for non-functional receptors. An increased diversity can then be achieved by rerandomization or shuffling by staggered extension process (SteP) of the existing library and further rounds of FACS sorting (Sarkar et al., 2008). This method has been employed to stabilize rat neurotensin receptor 1, α_1a_-adrenergic receptor, α_1b_-adrenergic receptor and tachykinin receptor NK1 (Sarkar et al., 2008; Dodevski and Plückthun, 2011).

An approach that results in a higher diversity of receptors is the generation of libraries for every amino acid position to be tested. For each library, a certain amino acid position is replaced by an NNN codon, which allows all 64 possible codons at this position (Schlinkmann et al., 2012b). The selection process is the same as for an error-prone PCR based library. Selected variants can then be combined by staggered extension process (StEP) or, for a complete coverage of all possible combinations, a new library containing all possible combinations can be generated using Slonomics® technology.

Another approach, cellular high-throughput encapsulation, solubilization and screening (CHESS), allows direct selection of detergent-solubilized mutants. A receptor library is expressed in *E. coli* cells and the cells are encapsulated with polymers leading to single-cell capsules each expressing a different receptor variant. The receptors are solubilized with a chosen detergent and incubated with a fluorescently-labeled ligand. Receptors retaining their function after detergent solubilization can then be selected by FACS (Scott and Plückthun, 2013; Scott et al., 2014). The expression vectors from selected cells are isolated, amplified and used for a further round of evolution. CHESS led to the most stable NTR1 variant reported to date; the construct termed NTR1-H4 showed a melting temperature of 57°C in presence of fluorescently labeled neurotensin while the variant generated by alanine scanning reached 43.7°C (Scott et al., 2014).

Directed evolution approaches with error-prone PCR have led to receptors expressing 2–18 times as many receptors compared to the wild-type GPCR. Higher initial expression levels (α_1b_-adrenergic receptor, twofold increase) could not be increased as much as very low initial expression levels (α_1a_-adrenergic receptor, 18-fold increase) (Sarkar et al., 2008; Dodevski and Plückthun, 2011). Combined approaches with initial improvement of rat neurotensin receptor 1 by error-prone PCR-based evolution led to a variant with a 12-fold higher expression level (Dodevski and Plückthun, 2011). This variant was improved to 50-fold increased expression compared to wild type using all-vs.-all mutations (Schlinkmann et al., 2012b).

**Figure 1:**
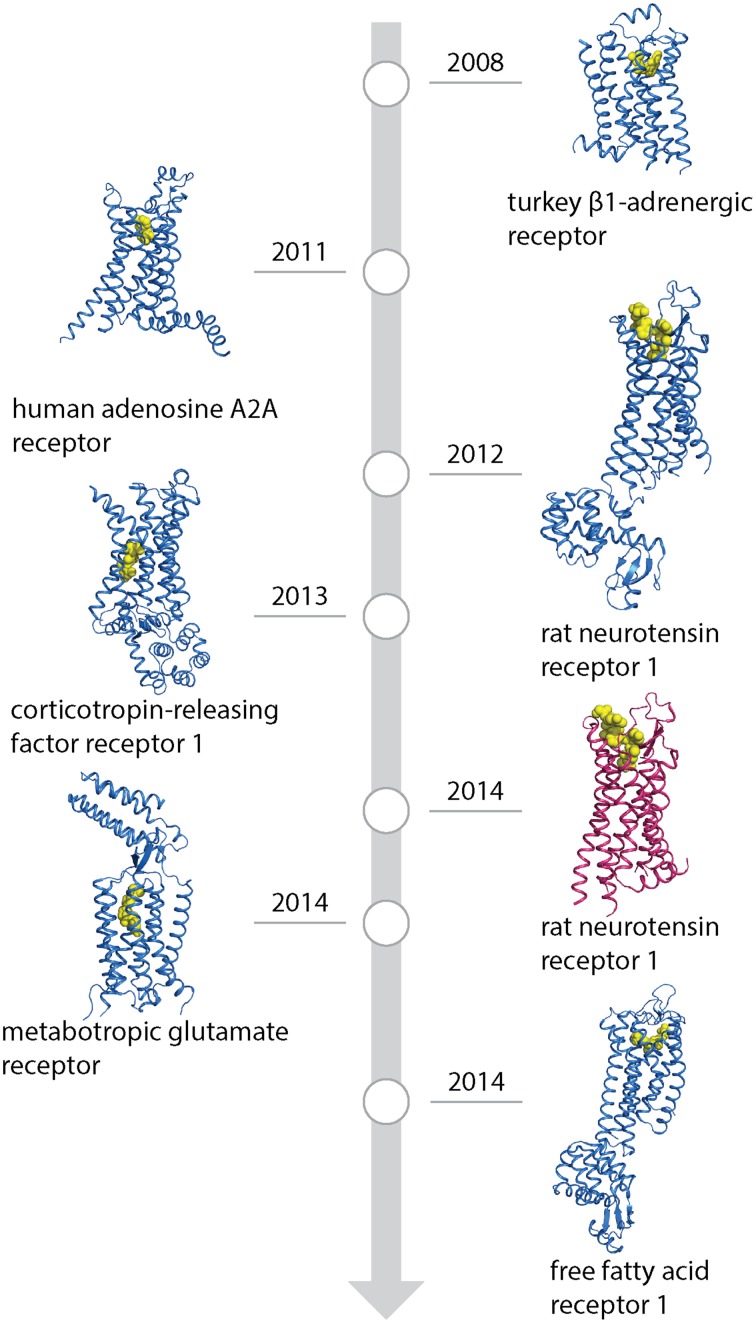
**Timeline of GPCR structures based on conformational thermostabilization by alanine scanning (blue) or directed evolution (red) with their ligands (yellow)**. PDB IDs: 2VT4 (turkey β1-adrenergic receptor), 2YDO (human adenosine A_2A_ receptor), 4GRV (neurotensin recetor 1, conformationally stabilized), 4K5Y (corticotropin-releasing factor receptor 1), 4BV0 (neurotensin receptor 1, directed evolution), 4OR2 (metabotropic glutamate receptor) and 4PHU (free fatty-acid receptor 1).

**Figure 2:**
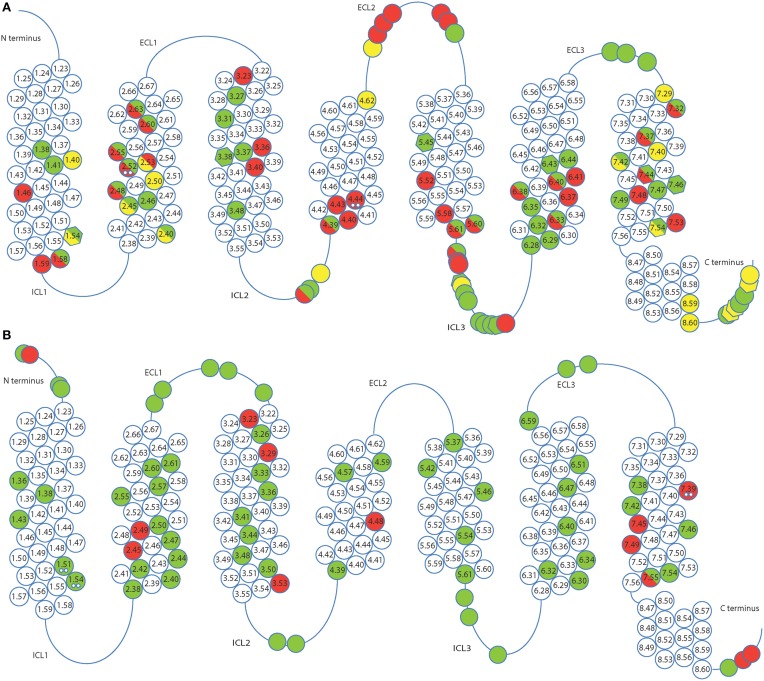
**Favorable mutations identified by alanine scanning (A) and directed evolution (B) in presence of agonist (green), antagonist (red) and in absence of ligand (yellow)**. Alanine scanning identified mutations which increase the thermostability of receptors while retaining a minimal expression level. Directed evolution detected mutations that increased expression or thermostability, or both. Position of mutations is indicated by Ballesteros-Weinstein numbering. Mutations found in multiple receptors are indicated by double dots, those with high expression levels (=125% of wild-type expression level, alanine scanning only) are shown as hexagons. ECL and ICL stand for extracellular and intracellular loops, respectively.

Structures solved after thermostabilization by alanine scanning or directed evolution are represented in Figure 1 and favorable mutations found in different receptors are shown in Figure 2 and Supplementary Table S1. Alanine scanning identified approximately 90 mutations and 70 were found using directed evolution. The identified mutations are distributed all over the receptor sequence, including both the transmembrane helices and loop regions. It is interesting to note that 15 mutations (ca. 10%) overlap between sets of mutations derived by two approaches. This number only refers to mutations in the transmembrane parts because sequence conservation of the loop regions is very weak, and, therefore, direct comparison of the residue positions in different receptors is not always possible. However, it has to be noted that a significant number of stabilizing mutations was identified in the loop regions (ca. 30%), as well as in the presumably unstructured C-terminus of the receptor at the positions after the predicted helix 8. Given that the majority of these mutations were identified in *E. coli* based screens which lacks proteins interacting with the receptors (e.g., arrestins), this strongly suggests that all of these positions are involved in stabilizing interactions and are in structured environments.

## Constitutively active mutants

For the majority of crystal structures, modified receptors with little basal activity were used. Most receptors were bound to inverse agonist or antagonist and correspond to receptors in their inactive state (Deupi and Standfuss, 2011). Constitutively active mutations, however, may conformationally stabilize the active state of the receptor and may be useful for structural studies. These mutants are ubiquitously found among GPCRs and are often related to different diseases (Schöneberg et al., 2004). Although multiple mechanisms may link a mutation to a disease, at least in some cases these mutations were shown to activate the receptor in the absence of the agonist. There are several examples of using constitutively active mutations for the structural studies of rhodopsin.

Mutations of the two amino acids in the ligand binding pocket, Lys296^7.42^ and Glu113^3.28^, lead to strong constitutive activation of opsin due to the perturbation of the salt bridge which stabilizes the inactive ground state (Cohen et al., 1992; Robinson et al., 1992; Standfuss et al., 2008). A crystal structure of E113^3.28^Q rhodopsin in complex with a peptide derived from the C-terminus of the alpha subunit of the G protein transducin (GαCT) represents an active state of rhodopsin (Standfuss et al., 2011). A structure of the disease-inducing mutation G90^2.56^D shows pertubations of the same Lys296^7.42^-Glu113^3.28^ salt bridge (Singhal et al., 2013). Constitutive activity of the M257^6.40^Y mutant most probably stems from a stabilization of the open G-protein binding pocket as suggested by its crystal structure (Deupi et al., 2012).

The constitutively active mutants have also been shown to be very promising for formation of the stable GPCR-G protein signaling complex. The E113^3.28^Q mutant forms a rhodopsin Rho-G-protein transducin (Gt) complex that is, opposite to the native complex, stable in detergents (Xie et al., 2011). The M257^6.40^Y mutant forms a very stable Rho-Gt and an even more stable Rho-Gi complex which is superior to the E113^3.28^Q complex for crystallization trials due to its higher detergent resistance and long-term stability (Maeda et al., 2014).

Overall, it is likely that constitutively active mutants will have a big impact on structure determination of signaling complexes of rhodopsin and other GPCRs.

## Computational approaches

The emergence of the experimental data on GPCR stabilization by mutagenesis, combined with crystallographic structures of GPCRs, allows for the rationalization of the effects of mutations on protein stability. More importantly, it allows for computation prediction of stabilizing mutations. A very interesting approach is to identify the metastable regions in the GPCRs, which are responsible for their conformational flexibility, and to stabilize these regions by improving side chain packing (Chen et al., 2012b). Using wild type and stabilized turkey β1-adrenergic receptor as starting points, the authors were able to predict and experimentally confirm several mutations which significantly increased the stability of both proteins. In addition of being able to identify novel stabilizing mutations, this method also rationalizes 70% of stabilizing mutations identified by alanine scanning of turkey β_1_-adrenergic and A_2A_ receptors. An alternative approach to identify stabilizing mutations is based on generation of the ensemble of conformations based on a homology model, and the prediction of an average enthalpy around the mutation site (Bhattacharya et al., 2014). This parameter was shown to correlate relatively well with the observed stabilization effect of mutations in turkey β1-adrenergic, A_2A_ and NTR1 receptors. As our understanding of the role of the individual amino acids in GPCR structure and stability improves, and more experimental data become available, the computational approaches to receptor design will likely become more powerful and widely used in the future.

## Methods for assessment of receptor stability

### Radio-ligand binding

Radio-ligand binding as described in the alanine scanning section is the most widely used high-throughput method for determination of stability, assessment of the percentage of receptor still active after incubation at a given temperature and comparison to the stability of the wild-type. One of the reasons for this is the sensitivity of the method, the corresponding very low sample requirements, and the ability to work with non-purified samples and even membranes. On the other hand, this method depends on the availability of the desired ligand labelled with either ^3^H or ^125^I.

### Fluorescence

Fluorescently-labeled ligand can be used for high-throughput stability tests in any given condition such as different detergent solutions. The remaining ligand fluorescence after incubation reflects how much receptor is still active after the incubation period. The ligand fluorescence can be normalized by measuring the fluorescence of a genetically-encoded fluorescent protein such as GFP. The assay set-up is facilitated by a purification tag which allows binding of the receptor to magnetic beads since they can be easily transferred to assay and washing solutions. The assay has been developed for neurotensin receptor 1 using HiLyte Fluor 647-labeled neurotensin, GFP-labeled NTR1 for receptor quantification and an Avi-tag for *in vivo* biotinylation and capture on streptavidin-coated magnetic beads (Scott and Plückthun, 2013; Scott et al., 2014).

### A fluorescence-detection size-exclusion chromatography-based thermostability assay (FSEC-TS)

Traditional methods that evaluate protein stability require large amounts of material, and are therefore ill-suited for medium-to-high-throughput screening of membrane proteins (Hattori et al., 2012). With this method the proteins can be analyzed in either purified or unpurified form (solubilized lysates) and it allows evaluating microgram to nanogram amounts of samples. Unpurified target proteins need to be expressed as a GFP fusion, while for the purified protein tryptophan fluorescence can be used. Purified or unpurified proteins are incubated over a range of temperatures and then applied to a size-exclusion chromatography column in line with a fluorescence detector to monitor GFP fluorescence from GFP-tagged proteins or tryptophan fluorescence from endogenous Trp residues (Hattori et al., 2012). The results provide an apparent melting temperature (T_m_), which can be used as a reference point to test the degree of protein thermostabilization. This method also allows testing of different ligands, ions, detergents on the thermostability of the receptor (Hattori et al., 2012). Several proteins had their T_m_ determined in this way, for example P2X4, GluCl (Hattori et al., 2012) as well as a number of mutated and non-mutated proteins in the authors' laboratory. This method has also been used for the determination of T_m_ of a complex RhoM257Y/Gi (Maeda et al., 2014). The main advantage is that it can be used for small amounts of unpurified sample, which saves time and sample that might be lost through the process of purification. However, in this case the protein needs to be fused to GFP and the throughput of the method is still relatively low compared to some other methods currently used in this field. While the experiments have been done mostly with GFP fusions so far, other fluorescent fusions can also be used (e.g., RFP, YFP).

### Differential scanning fluorimetry (DSF)

A faster method for determination of melting temperatures is differential scanning fluorimetry (DSF) using 7-diethylamino-3-(4′-maleimidylphenyl)-4-methylcoumarin (CPM) dye (Alexandrov et al., 2008). CPM dye is a thiol-reactive probe which reacts with cysteines once they are exposed. Upon binding to a cysteine, the dye becomes fluorescent. Increasing fluorescence is therefore a measure of protein unfolding. The method can be performed in either a fluorescence spectrometer or, for a higher throughput, in an RT-PCR machine equipped with the respective filters (365 nm excitation, 460 nm detection). The method is fast and effective and needs small (1–4 μg) amounts of purified protein containing reduced buried cysteines.

### Homogeneous time resolved fluorescence

A very promising alternative to the radio-ligand binding stability assay is an assay based on homogeneous time resolved fluorescence (HTRF®) (Degorce et al., 2009) between a conformationally-specific 2D7 antibody recognizing the extracellular loop 2 (ECL2) of the CCR5 receptor, labeled with a Eu^3+^-cryptate donor, and a 1D4 antibody labeled with XL665 acceptor, targeted to the C-terminus of the receptor. Dissociation of the 2D7 antibody upon protein unfolding resulted in a decrease of the HTRF signal (Knepp et al., 2011). This assay allowed to measure protein stability change in the presence of a variety of ligands, and its sensitivity is comparable to radio-ligand binding assays.

### Other biophysical methods

If the purified receptor is available in reasonable amounts (100 μg to mg scale) more conventional biophysical methods can be applied. Since GPCRs have large alpha-helical content, circular dichroism (CD) spectroscopy will be sensitive to protein unfolding, and has been used to characterize olfactory receptor stability (Cook et al., 2009; Corin et al., 2011).

Intrinsic tryptophan fluorescence has been used to determine the thermostability of GPCRs in the authors' laboratory and can be used to measure their resistance to chemical denaturation (Ross et al., 2015).

### Chemical denaturation

Chemical denaturation of GPCRs offers a convenient alternative to temperature induced unfolding. To induce unfolding, the concentration of the denaturing agent, such as urea, guanidinium chloride or harsh detergents such as SDS is gradually increased (Zhou and Bowie, 2000; Zhou et al., 2001; Sehgal and Otzen, 2006; McKibbin et al., 2009; Miller et al., 2009; Curnow et al., 2011; Harris et al., 2014; Tastan et al., 2014).

For example, it is possible to deduce the binding constant of a ligand to protein from the concentration dependence of ligand- induced stabilization. Because chemical denaturation is performed at a constant temperature, the measurement of the K_d_ is straightforward (Ross et al., 2015).

## Molecular basis for stabilization

**Figure 3:**
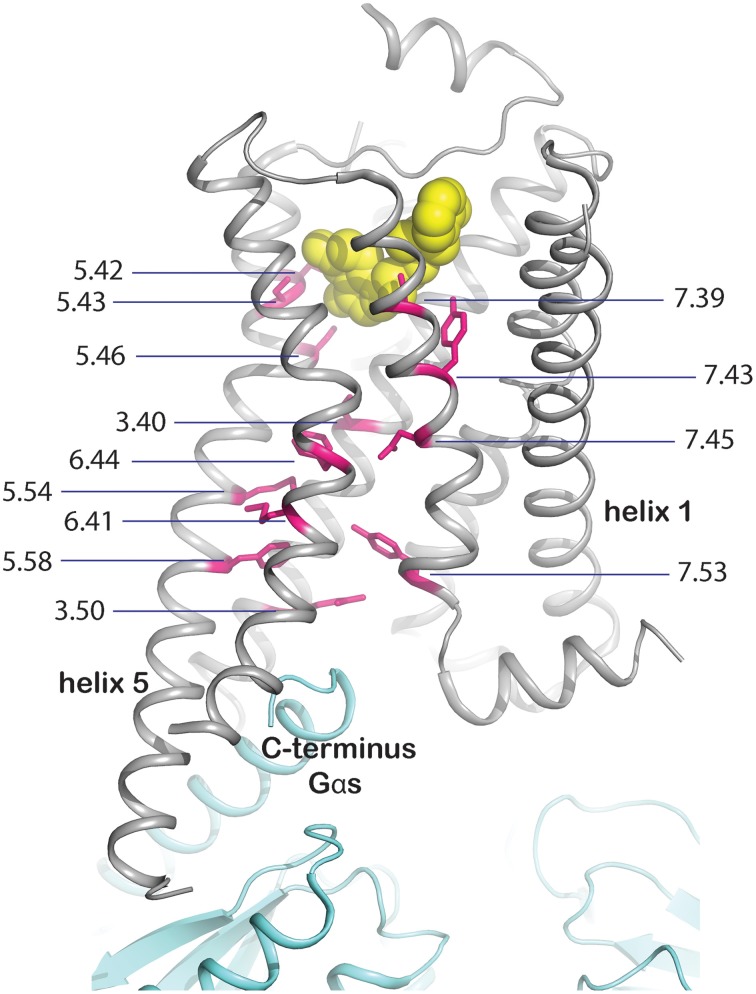
**Molecular basis for stabilization by mutation of residues involved in activation**. Residues directly involved in GPCR activation (shown as magenta sticks with Ballersteros-Weinstein numbers and the backbone shown in gray), were identified by comparison of inactive and active state structures. They form a continuous path between the ligand (yellow spheres) and the effector binding site, mapped on the crystal structure of the complex between human β_2_adrenergic receptor and the heterotrimeric Gs (cyan, PBD ID 3SN6, Rasmussen et al., 2011b).

Based on our comparison of inactive and active structures and the current knowledge of the structural basis of GPCR activation we suggest a classification of stabilizing mutations into four main groups:

- Mutation of residues directly involved in the activation mechanism. This group comprises the mutation of residues which are responsible for stabilizing the active state upon agonist binding and were identified by comparison of active and inactive-state structures. These residues are oriented toward the core of the transmembrane bundle and create a continuous path between the ligand binding site and the transducer binding site (Figure 3, Rasmussen et al., 2011b). We hypothesize that mutation of these residues may stabilize a particular, either active or inactive, state of the receptor. The positions in the activation path may be used to design conformationally stabilizing mutations for the inactive or active state. Several mutations in these positions have been identified by alanine scanning or directed evolution approaches (Table 1). Mutation of these residues may modify the local energy minima of the inactive and active states, and/or the energy barrier between them. This would alter the equilibrium between the states, stabilizing a specific conformation. A representative example of this group is Y227^5.58^A in turkey β_1_AR (Serrano-Vega et al., 2008). This tyrosine is important for the activation mechanism of GPCRs (Goncalves et al., 2010; Dror et al., 2011) as in the active state it interacts with R^3.50^ and with Y^7.53^ through a water-mediated hydrogen bond (Deupi et al., 2012), stabilizing the outward movement of helix 6. In rhodopsin, mutation of Y^5.58^ to F leads to a less stable active state (Goncalves et al., 2010). Similarly, its mutation to alanine in the β1AR may destabilize the active conformation of the receptor, by removing the possibility of interaction with R^3.50^ and Y^7.53^, resulting in the observed stabilization of the inactive state.
- Mutation of residues that are indirectly involved in the activation process. These residues would modulate the conformation of the residues from the first group either through direct interactions or by influencing specific inter-helical packing regions that affect the relative energy between the side chain conformations of the residues in the first group. A representative example is L310^6.37^A in NTR1 (White et al., 2012; Shibata et al., 2013). A leucine at this position places a bulky side chain between R^3.50^ and N^5.58^ (as observed in the structure of many inactive GPCRs; approximately 80% of Class A GPCRs have L/V/I at this position). Mutation of this residue to alanine in the NTR1 alters the packing between transmembrane helix 3 (TM3), TM5 and TM6 by reducing the volume of the side chain. As a result, in the structure of the thermostabilized NTR1 (White et al., 2012), R^3.50^ features a “warped” conformation (possibly aided by the neighboring mutation E166A^3.49^) that may reduce the energy barrier for its interaction with N^5.58^, stabilizing an active-like structure.
- Mutation of residues facing lipids or detergent. These mutations may influence the properties of the outer surface of the receptor and change its interaction with the lipid and detergent molecules. A representative example is F338^7.48^M in tβ_1_AR (Serrano-Vega et al., 2008). In this case, a rigid phenylalanine is replaced by the flexible side chain of methionine which complements A^7.44^ one turn above, resulting in a smoother surface to which lipids or detergent may adjust with higher compatibility.
- Mutation of residues responsible for local structural stability. These residues are located, for instance, in the interface between helices, or in the interface between helices and loops, and their mutation may alter the structural or dynamic properties of the local secondary structure. A representative mutation is I55^1.46^A in tβ_1_AR (Serrano-Vega et al., 2008). This residue is located in the interface between TM1, TM2, and TM7, and most GPCRs (approx. 90%) have a small residue (G/A/S/T) in this position. In the crystal structures of receptors with a small side chain at this position (e.g. CCR5, PAR1, μ-OR), TM1 is closer to the rest of the transmembrane bundle than when this residue contains a bulky side chain (e.g., β_1_AR, β_2_AR, S1P1R). Thus, mutation of this residue to alanine in the tβ_1_AR might allow TM1 to come closer to the transmembrane bundle and improve the packing with TM2 and TM7.

**Table 1:** **Mutations identified in the activation path of class A GPCRs**.

**Ballesteros-Weinstein number**	**Receptor**	**Ligand type**	**Mutation**	**Identified by**
3.40	Turkey β1-adrenergic receptor	Antagonist	I129V	Alanine scanning
3.50	Rat neurotensin receptor 1	Agonist	R167L	Directed evolution
5.42	Tachykinin receptor 1	Agonist	C199G	Directed evolution
	Rat neurotensin receptor 1	Agonist	V240L	Directed evolution
5.43	-	-	-	-
5.46	Tachykinin receptor 1	Agonist	I204T	Directed evolution
5.54	Rat neurotensin receptor 1	Agonist	I253A	Directed evolution
5.58	Turkey β1-adrenergic receptor	Antagonist	Y227A	Alanine scanning
6.41	Adenosine A_2A_ receptor	Antagonist	V239A	Alanine scanning
6.44	Adenosine A_2A_ receptor	Agonist	F242A	Alanine scanning
7.39	α_1A_-adrenergic receptor	Antagonist	F312L	Directed evolution
	α_1B_-adrenergic receptor	Antagonist	F334L	Directed evolution
7.43	Turkey β1-adrenergic receptor	Antagonist	A334L	Alanine scanning
7.45	α_1A_-adrenergic receptor	Antagonist	N318H	Directed evolution
7.53	Turkey β1-adrenergic receptor	Antagonist	Y343L	Alanine scanning

*These mutations may stabilize an agonist or an antagonist-bound state of the receptor*.

## Rational design and transferability of mutations

Most thermostabilizing point mutations found in alanine scanning approaches have been shown to stabilize either the inactive or the active state. The two sets of mutations found for human adenosine A_2A_ receptor using either the agonist 5′-*N*-ethylcarboxamidoadenosine (NECA) or the antagonist ZM241385 contain 27 and 17 mutations, respectively. Only three mutations were found to stabilize both conformations (Magnani et al., 2008). This shows that the mutations found depend largely on the ligand used for the stability assay, and, correspondingly, on the conformation of the receptor. The sets of mutations found for human adenosine A_2A_ receptor, turkey β1-adrenergic receptor and rat neurotensin receptor NTR1 do not overlap; transfer of these mutations to other GPCRs might not lead to a stabilization of the unliganded state but could have a stabilizing effect on the respective liganded receptors.

**Figure 4:**
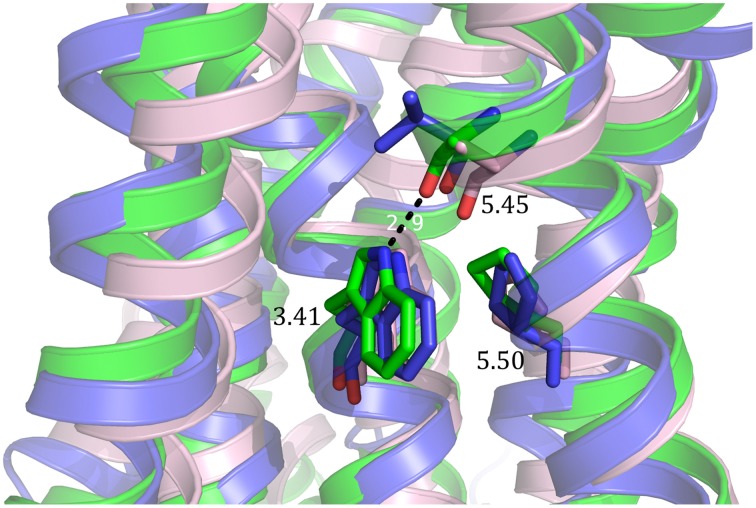
**Molecular basis of stabilization by mutation of the 3.41 position to tryptophan**. The 3.41W mutation stabilizes 5-HT1B (green), 5-HT2B (light pink) and CXCR4 (blue) through its interaction with the proline in position 5.50 and the carbonyl of the amino acid in position 5.45.

One mutation, E122^3.41^W, identified in human β2-adrenergic receptor has been shown to be transferable to different GPCRs and increased thermostability as well as total expression level and expression at the cell surface (Roth et al., 2008). In rhodopsin, W126^3.41^ is the only residue in transmembrane (TM) domain 3 which contacts both TM4 and TM5. Since position 3.41 is not conserved, introduction of a mutation is less likely to disturb the overall fold of the receptor. The tryptophan at the TM3-TM4-TM5 interface is thought to stabilize the conformationally flexible TM5, and thereby increase thermostability and expression. The effect on β1-adrenergic receptor was similar to thermostabilization by addition of antagonist (Roth et al., 2008). Other mutations of the E122^3.41^ residue also led to higher thermostability and expression, especially E122Y and E122L. However, all mutations showed a loss in affinity for the ligand. The mutation which showed the highest increase in thermal stability has been successfully transferred to the serotonin receptors 5-HT_1B_ (L138W) and 5-HT_2B_ (M144W) as well as the CXCR4 chemokine receptor (L125W) (Wu et al., 2010; Wacker et al., 2013; Wang et al., 2013) and D3 dopamine receptor (L119W) (Chien et al., 2010). As proposed by molecular modeling of human β2-adrenergic receptor (hβ_2_AR), W^3.41^ stabilizes 5-HT_1B_, 5-HT_2B_ and CXCR4 by its interaction with P^5.50^ and the carbonyl of 5.46 (Figure 4). Since conformational flexibility of TM5 is an inherent feature of class A GPCRs, transfer of this mutation is likely to be successful (Roth et al., 2008).

About 90 thermostabilizing mutations were identified in GPCRs by alanine scanning so far, however, only two of these mutations were predicted to be transferable to other receptors (Serrano-Vega and Tate, 2009). The transferability of the mutations has been tested on adrenergic receptors. Turkey β_1_AR (tβ_1_AR) is 76% and 59% identical to human β_1_AR (hβ_1_AR) and β_2_AR (hβ_2_AR) respectively (Serrano-Vega and Tate, 2009). For the comparison N- and C-termini were removed. All six thermostabilizing mutations (referred to as the m23 mutations: R68S, M90V, Y227A, A282L, F327A, and F338M) that were previously determined in tβ_1_AR were transferred *en bloc* to the human receptors. The initial test of the thermostability of non-mutated proteins showed that both β1 receptors prefer a lipid-rich environment rather than just DDM, while hβ2AR is largely insensitive to different DDM concentrations (Serrano-Vega and Tate, 2009). Melting temperatures of the receptors with mutations were increased by 21°C, 17°C, and 11°C for tβ_1_AR, hβ_1_AR, and hβ_2_AR, respectively. Since the optimal amino acid residues required to stabilize the human receptors could be different from those that stabilized turkey receptor, different residues were tried at the six positions. While the majority of the mutants showed similar or worse thermostability compared to the original m23 mutants, some exceptions were found (Serrano-Vega and Tate, 2009). The m23 thermostabilizing mutations preferentially stabilized the turkey receptor in the antagonist bound conformation and data show that they are affecting the conformation of human receptors in a similar way (Serrano-Vega and Tate, 2009).

Generally, transfer of other mutations could be a possibility for other closely related receptors or for mutations of residues which show a high degree of identity or similarity between different receptors.

## Does stabilization alter the structure of receptors?

**Figure 5:**
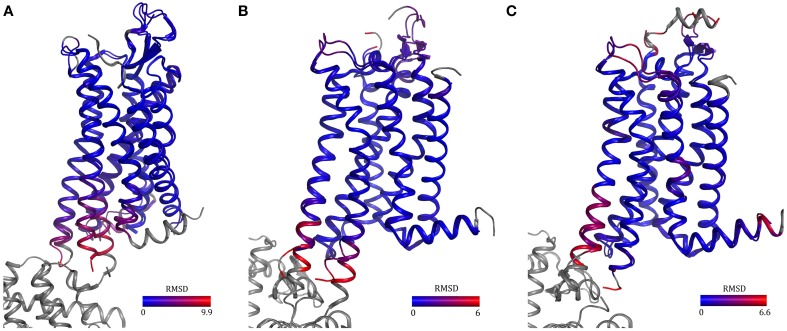
**Comparison of GPCRs containing either a fusion protein or thermostabilizing mutations**. Structures of neurotensin receptor 1 **(A)** and adenosine A_2A_ receptor in the inactive **(B)** and active **(C)** state solved using fusion-protein or point-mutagenesis approaches show high similarity [overall RMSD = 0.95 Å **(A)** and 0.5 Å **(B,C)**]. The differences in helices five and six in the two neurotensin receptor structures may be due to mutation of an amino acid involved in activation, R167^3.50^L, in one receptor and the T4 lysozyme fusion in the other receptor. PDB IDs: 3ZEV (NTR1, directed evolution), 4GRV (NTR1, fusion with T4 lysozyme), 3PWH (A_2A_, antagonist-bound, with thermostabilizing mutations), 3EML (A_2A_, antagonist-bound, T4 lysozyme fusion), 3QAK (A_2A_, with agonist UK-432097 and T4 lysozyme) and 2YDO (with agonist adenosine and thermostabilizing mutations).

Two rat neurotensin receptor 1 variants selected by CHESS and a variant harboring 11 mutations uncovered by recombination and evolution of position-specific libraries have been crystallized (Schlinkmann et al., 2012a,b; Egloff et al., 2014). All three structures are almost identical even though the sets of mutations are different (overall RMSD = 0.4Å). In addition, one variant was competent of G-protein activation, though at a reduced level, ligand binding with native-like affinities and desensitization. The structure looks like an inactive agonist-bound conformation since TM6 did not move outwards, which agrees well with the reduced G-protein activation observed and the fact that R167^3.50^, a residue involved in the activation mechanism has been mutated to leucine. The T4L structure of NTR1 (White et al., 2012, PDB ID 4GRV), supposedly in a semi-active conformation, is very similar to the structures obtained by directed evolution. They only differ at the intracellular ends of TM5 and 6 which might be due to different degrees of activation and the fusion with T4L (Figure 5A).

Two versions of turkey β1 adrenergic receptor, m23 and JM50 (PDB IDs 2VT4 and 4BVN), differ by three additional mutations in the JM50 construct (Warne et al., 2008; Miller-Gallacher et al., 2014). I129^3.40^V, a mutation in the transmembrane core, does not alter the structure when compared to m23. D322K and Y343L which are in the extracellular loop 3 and at the intracellular end of TM7, respectively, change the structure by 1.5–2 Å. This change is probably due to reduced dynamic properties of regions which are generally more flexible. It can be concluded that the structural influence of these mutations is marginal.

Three versions of human adenosine A_2A_ receptor with conformationally thermostabilizing mutations, T4L- or bRIL-fusion (PDB IDs 3PWH, 3EML, and 4EIY) and with the same inverse agonist bound show very similar structures, differences are in flexible parts only which means that the structure is not changed by the stabilizing mutations (Figure 5B). One difference is a small change at the intracellular side of TM5 which might be due to fusion partners. The agonist-bound A_2A_ structures (PDB IDs 3QAK and 2YDO) show high similarity, the biggest difference is found in the residues contacting the ligand. This is due to ligand differences—additional bulky groups—which push away ECL3 and the extracellular part of TM7 in the 3QAK structure. Additional differences, also caused by the ligand dissimilarities, occur in one turn of TM5 (C185^5.46^), which is pushed away by 1.6Å in the 3QAK structure. Lack of ECL2 in the 3QAK structure makes this region impossible to compare. The overall RMSD of these two structures is 0.5 Å (Figure 5C) (Lebon et al., 2011b; Xu et al., 2011).

Receptors can additionally be stabilized by ligands, which was extensively studied for turkey β1 adrenergic receptor (Warne et al., 2011). The thermostabilized receptor was preferentially in the inactive state, but could still couple to G proteins after activation by agonist, although the activation energy barrier is predicted to be considerably higher than for the wild-type receptor. All determined structures are very similar to the one determined with the bound antagonist cyanopindolol as expected for a receptor mutant stabilized preferentially in the inactive state. Agonist binding, however, induced a 1 Å contraction of the catecholamine binding pocket relative to the structure with bound antagonist (Warne et al., 2011). Overall, insertion of mutations does not seem to alter GPCR structures; the published structures of adenosine A_2A_ and neurotensin receptor 1 with and without stabilizing mutations show high similarity (RMSD = 0.5 Å).

## Pharmacological effects of mutations on turkey β1-adrenergic receptor

The pharmacological effects of stabilization have been extensively studied for thermostabilized turkey β1-adrenergic receptor. It was shown that deletions of N- and C-termini as well as residues in the loops (β36 deletions, Warne et al., 2009) did not affect ligand binding which is consistent with the view that the N- and C-terminus and most of cytoplasmic loop 3 do not interact with the ligand-binding pocket (Baker et al., 2011). Insertion of thermostabilizing mutations, however, reduced the affinity of all ligands approximately tenfold with some of the most efficacious agonists showing an even higher reduction. Previous ligand binding data (Serrano-Vega et al., 2008) showed that cyanopindolol and dihydroalprenolol bound to both versions of the receptor with similar affinities, while binding of the agonists isoprenaline and noradrenaline was reduced dramatically. This difference in ligand binding behavior is likely to be related to the differences in the environment both sets of the experiments were performed in (Serrano-Vega et al., 2008; Baker et al., 2011). Isoprenaline, adrenaline and noradrenaline were able to stimulate activation of thermostabilized turkey β1-adrenergic receptor though much higher concentrations were needed to achieve the response in the thermostabilized version (Baker et al., 2011).

## Properties and uses of stabilized GPCRs

While structure determination of GPCRs with fusion proteins required special crystallization techniques, namely lipidic cubic phase (LCP) crystallization, the structures of conformationally thermostabilized or evolved receptors could be determined using crystals obtained in vapor diffusion set-ups. In addition, crystal structures of receptors bound to weak ligands were only obtained with conformationally thermostabilized receptors so far (Congreve et al., 2014). Conformational thermostabilization increases receptor homogeneity and stability in a wider range of detergents which in turn facilitates crystallization (Congreve and Marshall, 2010).

One of the main problems for biophysical characterization of GPCRs is their inherent instability, loss of native structure upon solubilization and low conformational homogeneity. Conformationally thermostabilized receptors, however, show high activity and conformational homogeneity which results in good quality data obtained from biophysical experiments such as surface plasmon resonance (SPR) even when small ligands are used (Rich et al., 2011). Binders can be rapidly identified and ranked according to their affinity and kinetics (Congreve et al., 2011). Furthermore, SPR can be used for biophysical mapping of the ligand binding site. Ligand-binding site residues are identified, mutated and screened vs. an array of structurally and pharmacologically different ligands. Since a thermostabilized receptor is used as a basis for mutations, high activity and native conformation are ensured. This approach gives insights on ligand interaction with the receptor and shows which residues affect binding of different ligands. These data can be used for identification of selective compounds in fragment-based drug discovery (Zhukov et al., 2011).

Fragment-based drug discovery with nuclear magnetic resonance (NMR) experiments have been hindered by the limited availability of receptor and the nonspecific partitioning of ligands into detergent micelles. In target-immobilized NMR screening (TINS), thermostabilized receptors solve the problem of availability of active receptor. (Congreve et al., 2011). Binding of mixtures of three to eight fragments of limited size (<300 Da) and low affinity (>1 μM) is then monitored with 1D ^1^H NMR spectra (Chen et al., 2012a). Since spin relaxation is far more efficient in unbound fragments, fragment binding can be detected by loss of signal.

A great advantage of conformationally thermostabilized receptors in fragment-based drug discovery is the prospective identification of fragments which selectively bind receptor active states and therefore development of functional agonist (Congreve et al., 2011).

Generation of therapeutic antibodies against GPCRs is often hindered by the lack of sufficient amounts of stable, homogeneous, native-state protein. Conformationally thermostabilization of GPCRs may present a very good tool for antibody generation, since the protein can be made in larger quantities and is stable in short-chain detergents which do not mask most relevant epitopes. Furthermore, the receptors can be stabilized in antagonist or agonist-bound forms which allows raising of antibodies against specific GPCR conformations (Hutchings et al., 2010).

## Conclusions

Conformational thermostabilization of many different receptors using alanine/leucine scanning or directed evolution has shown that point mutations are a valuable tool for GPCR structural biology and biophysics. A relatively large fraction (5–12%) of alanine mutations in scanning mutagenesis led to the stabilization of the receptors, suggesting that it is likely that many receptors could be stabilized by this approach.

Stabilization by point mutations in combination with the truncation of the flexible parts of the receptor has led to the successful crystallization and structure determination of several receptors including human adenosine A_2A_ receptor, rat neurotensin receptor 1 and turkey β1-adrenergic receptor. The stabilizing mutations can also be combined with a fusion partner approach therefore expanding the repertoire of approaches which can be used to obtain receptor structure. Designed mutations facilitated crystallization of human β2-adrenergic receptor, serotonin receptors 5-HT_1B_ and 5-HT_2B_ as well as chemokine receptor CXCR4 and constitutively active mutations found for many diseases may facilitate structure determination of active-state GPCRs or their G-protein complexes. Addition of mutations is certainly a very powerful technique which can complement all other techniques to stabilize the receptors for structural and biophysical characterization.

The exact molecular reasons for why a particular mutation is stabilizing are not always obvious. In this work, we proposed four categories of stabilizing mutations based on their role in the protein ranging from direct to indirect contribution to the activation mechanism to local stabilization of the structure and facilitate interactions with the lipid or detergent environment. The classification certainly furthers our understanding of the molecular basis for stabilization by mutations and may help in predicting mutations in the future.

One might hope that in the future it might be possible to design thermostabilizing mutations, computationally predict them or transfer them from other receptors, or that the development of technology would reduce the effort of finding stabilizing mutations to the point that it would become a widely used approach in GPCR and other unstable dynamic proteins structural and biophysical studies.

### Conflict of interest statement

The authors declare that the research was conducted in the absence of any commercial or financial relationships that could be construed as a potential conflict of interest.

## Supplementary material

Supplementary Table 1 **Favorable Mutations identified in GPCRs**.
